# Researches on Hydrophobia

**Published:** 1885-02

**Authors:** 


*Researches on Hydrophobia.*—Pasteur, Chamberland and

Roux have communicated to the Academy of Sciences of Paris the important results of their studies.

The great fact of the variable virulence of one virus, and the preservation of a virulence for another of less intensity, is now an acquired fact of science and practice.

The virulence of the virus of rabies becomes enfeebled by the passage from the dog to a monkey and from the monkey to another monkey, and if the virus so enfeebled is carried back to the dog, to the rabbit and to the guinea pig, it is again attenuated. By the passages from the monkey to a monkey the attenuation can be brought to a condition unable to produce the rage in the dog, nor by the method of hypodermic inoculations, nor by the surer one of the trepanation.

The virulence of the said virus increases, if passed from rabbit to rabbit, or from guinea pig to guinea pig, and when it is carried to a maximum in the rabbit, it is transmitted in such a condition to the dog as to produce a rage always mortal.

It will now be of great interest to be able to suppress the development of hydrophobia following bites of rabid dogs.

Pasteur believes that, on account of the long period of incubation, the refractory state of the bitten subjects can be determined with a surety before the disease has manifested itself. The first attempts gave the greatest hopes of success, but it is necessary that they be multiplied *ad infinitum* and on different species of animals before they be attempted on man.—*Le Progrès Medical.*

